# SbHsp70 overexpression enhances drought and salinity tolerance in wheat through improved cellular stability and stress-associated structural adaptations

**DOI:** 10.3389/fpls.2026.1868690

**Published:** 2026-06-18

**Authors:** Sruthy Maria Augustine, Nayyer Abdollahi Sisi, Lennart Scheer, Annabelle Heid, Babette Knoblauch, Stjepan Vukasovic, Stavros Tzigos, Rod J Snowdon

**Affiliations:** Department of Plant breeding, IFZ Research Center for Biosystems, Land Use and Nutrition, Justus Liebig University, Giessen, Germany

**Keywords:** biolistics, bobwhite, durum wheat, heat shock protein, Hsp70, interlocking marginal lobe, Kofa

## Abstract

**Introduction:**

Drought and salinity are major constraints to wheat productivity worldwide. Heat shock protein 70 (*Hsp70*) is a conserved molecular chaperone implicated in plant stress responses, but its role in cellular stability and structural adaptation in wheat remains poorly understood.

**Methods:**

To investigate its function, the sorghum-derived *SbHsp70* gene was constitutively overexpressed in durum wheat (cv. Kofa) and bread wheat (cv. Bobwhite) via particle bombardment. Transgenic lines were evaluated under controlled drought and salinity stress conditions using physiological, cellular, molecular, and agronomic analyses.

**Results:**

*SbHsp70* overexpression enhanced drought and salinity tolerance in both wheat backgrounds. Transgenic plants maintained higher membrane stability, relative water content, and photosynthetic activity under stress. Enhanced interlocking marginal lobe formation, altered actin organization, and modulation of stress-responsive gene expression were also observed. Importantly, transgenic lines maintained agronomic performance under drought without yield penalties under well-watered conditions.

**Discussion:**

These findings suggest that *SbHsp70* contributes to abiotic stress tolerance through improved cellular stability and stress-associated structural adaptations. While the observed cytoskeletal and transcriptional changes indicate a coordinated stress response, further studies are required to elucidate the underlying molecular mechanisms.

## 1 Introduction

Durum wheat (*Triticum durum*) is a globally important cereal crop valued for its high protein content and its primary use in pasta production, contributing significantly to food security in many regions of the world (Grosse-Heilmann et al., 2024). It is predominantly cultivated in arid and semi-arid environments, where productivity is increasingly constrained by abiotic stresses, particularly drought and salinity. The frequency and severity of these stresses are projected to intensify under ongoing climate change, posing a major threat to sustainable wheat production. Consequently, developing wheat varieties with enhanced tolerance to water deficit and soil salinity is essential for maintaining stable yields and ensuring long-term food and feed security (Aghaie and Tafreshi, 2020). Plants cope with abiotic stress through coordinated physiological adjustments and molecular defense mechanisms that safeguard cellular integrity (Sato et al., 2024). These responses include maintenance of membrane stability, regulation of water status, preservation of photosynthetic capacity, and activation of stress-responsive proteins. Among these protective systems, heat shock proteins (HSPs) play a central role. HSPs function as molecular chaperones that stabilize proteins, prevent stress-induced aggregation, and facilitate recovery of cellular processes following stress exposure (Mayer and Bukau, 2005; Song et al., 2025). In addition to protein folding, several HSPs have been implicated in membrane stabilization, modulation of stress signaling pathways, and mitigation of oxidative damage (Aghaie and Tafreshi, 2020).

Heat Shock Protein 70 (*HSP70*) is one of the most conserved and functionally versatile members of the HSP family. Beyond its classical chaperone activity, HSP70 has been associated with maintaining membrane integrity, protecting photosynthetic machinery, and regulating stress-adaptive cellular processes. Overexpression of *HSP70* genes has improved tolerance to drought, salinity, and temperature stress in multiple plant species, including Arabidopsis, rice, sugarcane, tobacco, and apple (Han et al., 2025; Liu et al., 2024; Kou et al., 2023; Berka et al., 2022; Jiang et al., 2020; Augustine et al., 2015b; Song et al., 2014). However, despite these findings, the mechanistic contribution of *HSP70* to physiological stability and structural protection in wheat, particularly under drought and salinity stress, remains incompletely characterized.

The *SbHsp70* gene used in this study was isolated from the drought-tolerant sorghum (*Sorghum bicolor*) genotype S579, which exhibits strong induction of *HSP70* under water-deficit conditions. Sorghum is widely recognized for its resilience to abiotic stress, and stress-adapted species represent valuable reservoirs of genetic resources for crop improvement. Introducing stress-responsive genes from such species into wheat may enhance intrinsic protection mechanisms while maintaining agronomic performance under favorable growth conditions. In the present study, we evaluated the effect of *SbHsp70* overexpression on drought and salinity tolerance in durum wheat and spring wheat. Transgenic lines were assessed under controlled stress conditions for physiological performance, membrane stability, water status, gas-exchange characteristics, and stress-induced cellular responses. By integrating molecular, physiological, and structural analyses, this work aims to clarify the functional role of *SbHsp70* in stress adaptation and to evaluate its potential as a genetic resource for developing climate-resilient wheat cultivars.

## 2 Materials and methods

### 2.1 Plant material and growth conditions

Durum wheat (*Triticum durum*) cultivars Antalis, Svevo, Kronos, Iride, Monastir, Meridiano, and Kofa were kindly provided by the University of Bologna (Italy). *Sorghum bicolor* genotypes 5282, Ji2732, Etios, and S579 were obtained from the germplasm collection of the Department of Plant Breeding, Justus Liebig University Giessen. Spring wheat (*T. aestivum*) cultivar Bobwhite was used for transformation experiments. Plants were grown in Fruhstorfer LD80 soil (HAWITA Gruppe GmbH, Germany) under controlled greenhouse conditions (22 ± 2 °C day/16 ± 2 °C night; 16 h light/8 h dark photoperiod; 60–70% relative humidity). Plants were maintained under well-watered conditions and fertilized biweekly with WUXAL Super NPK fertilizer until stress treatments were imposed.

### 2.2 Drought stress treatment

Drought stress was applied at the flowering stage by withholding irrigation for 10 days. Control plants were maintained under optimal irrigation. For recovery treatments, irrigation was resumed on day 11 and maintained thereafter. Experiments were conducted in a completely randomized design with five biological replicates per genotype per treatment.

### 2.3 Cell membrane thermostability analysis

Cell membrane stability was assessed following the method of Martineau et al. (1979) with minor modifications. Fully expanded third leaves were collected before stress induction (day 0) and after 10 days of drought treatment. Leaf discs measuring 0.5 cm in diameter and weighing approximately 200 mg were washed three times with 20 mL of distilled water, with each wash lasting 1 minute. After washing, 20 mL of distilled water was added to each control and treatment tube (2.5 cm × 15 cm). The tubes were sealed with aluminium foil and incubated at 60 °C in a thermostatically controlled water bath for 20 minutes. Both sets of tubes were then transferred to 10 °C for 12 hours to allow electrolytes to diffuse into the solution. Initial conductivity was measured at 30 °C. The tubes were subsequently heated to 100 °C for 20 minutes, after which final conductance was measured following cooling. The percentage of membrane injury is calculated using the formula:


$$\text{Membrane injury}\%=1-\left[\left(1-\text{T}1/\text{T}2)/(1-\text{C}1/\text{C}2\right)\right]\times 100.$$

where T and C represent the values for the treatment and control samples, respectively, and subscripts 1 and 2 denote the initial and final conductivity measurements.

### 2.4 Leaf water status or relative water content

Leaf relative water content (RWC) was determined using fully expanded third leaves sampled at day 0 and day 10 of drought treatment. Fresh weight (FW) was recorded immediately after sampling. Samples were hydrated to determine turgid weight (TW) and subsequently oven-dried to constant weight to obtain dry weight (DW). RWC was calculated according to Barrs and Weatherley (1962):


$$\text{RWC}\left(\%\right)=\left[\left(\text{FW}-\text{DW})/(\text{TW}-\text{DW}\right)\right]\times 100$$

### 2.5 Gas-exchange parameters and chlorophyll measurements

Photosynthetic rate (A), stomatal conductance (g s), and transpiration rate (E), were measured on the central region of the fully expanded third leaf on the 0^th^ (immediately before stress induction) and 10^th^ day of drought induction using a LI-6400 portable photosynthesis system (Li-COR Inc., USA). All measurements were recorded at a leaf temperature of 22 ± 2.0 °C, with data collected at 30-second intervals over a 3-hour period from 9:00 a.m. to 12:00 p.m. Chlorophyll content index (CCI) was measured using a CCM-200 chlorophyll meter (Opti-Sciences, USA).

### 2.6 Microscopy

Cellular morphological changes were examined in lower epidermal tissues of selected durum wheat cultivars, *S. bicolor* (S579), and *SbHsp70*-overexpressing Kofa and Bobwhite lines, along with their corresponding wild types. Actin microfilaments were visualized using a confocal microscope following phalloidin staining, adhering to the protocol outlined by Kobayashi et al. (1997) with minor alterations (Opalski et al., 2005). Sample fixation, permeabilization, and staining procedures were conducted according to established protocols with minor modifications (Kobayashi et al., 1997; Opalski et al., 2005). Representative images were obtained from at least three independent biological replicates per genotype and treatment.

### 2.7 Gene expression analysis using the comparative CT method

Total RNA was extracted separately from the 3rd fully opened leaf of each plant using RNAsolv reagent (Omega Bio-Tek), and genomic DNA contamination was removed by DNase I treatment. First-strand cDNA was synthesized using the RevertAid First-Strand cDNA Synthesis Kit (Thermo Fisher Scientific). Quantitative real-time PCR (qPCR) was performed using gene-specific primers (**Supplementary Table 3**) and SYBR Green on a StepOne Real-Time PCR System (Applied Biosystems). ADP-ribosylation factor was used as the internal reference gene after experimental validation of its stability under drought conditions. Relative expression levels were calculated using the 2^−ΔΔCt method (Livak and Schmittgen, 2001). Each reaction was performed in triplicate, with three biological replicates per genotype.

### 2.8 Vector construction and plant transformation

The *SbHsp70* coding sequence was amplified from *S. bicolor* (S579) and cloned under the control of the Port Ubi882 promoter (Phillip et al., 2013) into the pLH-6000-GFP vector (Imani et al., 2011). The primer details are given **Supplementary Table 1**. Particle bombardment-mediated transformation was performed using immature embryos (approximately 14 days post-anthesis) of durum wheat (Kofa) and spring wheat (Bobwhite). The transformation procedure followed the protocol described previously in (Sparks and Doherty, 2020). Regenerated plants were selected on hygromycin-containing medium (50 mg L⁻¹). Transgenic integration was confirmed by PCR analysis. Transgene copy number was estimated using quantitative real-time PCR (qPCR) by comparing the amplification of the transgene with that of the endogenous reference gene *HD1*. The *HD1* gene is present as two copies in the tetraploid wheat cultivar Kofa (AABB) and three copies in the hexaploid wheat cultivar Bobwhite (AABBDD).

qPCR reactions were performed using gene-specific primers for HD1 and promoter–transgene junction primers for *SbHsp70*. The primer sequences are given in **Supplementary Table 4**. Amplification efficiencies of both primer sets were validated and found to be comparable.

The relative transgene copy number was calculated using the comparative Ct method:

For Kofa:


$$\text{Copy number}=2\times 2^{-\left(Ct_{\text{transgene}}-Ct_{\text{HD}1}\right)}$$

For Bobwhite:


$$\text{Copy number}=3\times 2^{-\left(Ct_{\text{transgene}}-Ct_{\text{HD}1}\right)}$$

### 2.9 Visual scoring

Leaf wilting was visually evaluated at day 0 and day 10 of drought treatment using a modified 1–4 scale (Engelbrecht et al., 2007), where 1 indicates no wilting and 4 indicates severe irreversible wilting. Mean scores were calculated from five biological replicates per genotype.

### 2.10 Seed germination test

To evaluate salinity tolerance at the germination stage, seeds from transgenic and wild-type plants were subjected to irrigation with NaCl solutions (0, 100, 200, and 300 mM) under controlled greenhouse conditions. Germination percentage was recorded 30 days after planting. Experiments were conducted with multiple biological replicates per genotype and treatment.

### 2.11 Analysis of transgenic wheat genotypes in DroughtSpotter

Whole-plant drought responses were evaluated using the DroughtSpotter^®^ gravimetric phenotyping platform (Phenospex, The Netherlands) at Justus Liebig University Giessen (Stahl et al., 2020). Plants were grown in 60 L containers (90 kg soil) under controlled conditions (23 ± 2 °C; 16 h light/8 h dark). Automated irrigation maintained defined field capacities. Three treatments were applied: (1) well-watered (60% field capacity), (2) drought stress followed by recovery, and (3) continuous drought stress (40% field capacity from flowering onward). Five plants per container were maintained in a completely randomized design. Selected *SbHsp70*-overexpressing lines (K7, K8, B1, B2) were compared with wild-type controls. A complete randomised design was employed to evaluate the selected transgenic drought-tolerant wheat varieties in comparison to the wild type control (**Supplementary Figure 7**).

### 2.12 Statistical analysis

Differences among genotypes within treatments were analyzed using one-way ANOVA followed by Tukey’s HSD test. Pairwise comparisons between transgenic lines and wild-type plants were performed using Student’s t-test. Statistical significance was set at P ≤ 0.05.

## 3 Results

### 3.1 Differential drought tolerance among durum wheat cultivars and S. bicolor genotypes

**Figure 1:**
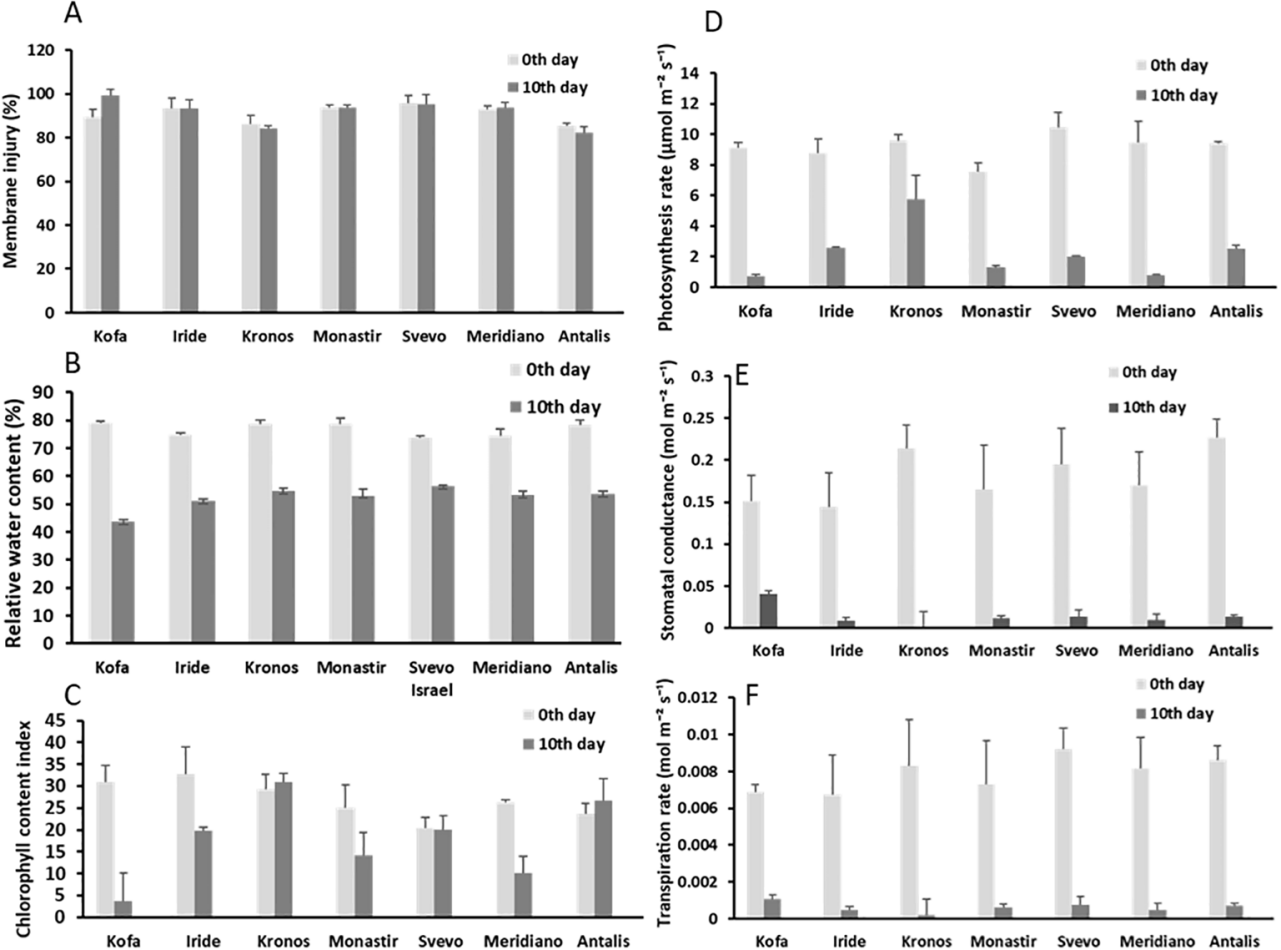
Physiological responses of wild-type durum wheat varieties under drought conditions. **(A)** Cell membrane thermostability, **(B)** relative water content (RWC), **(C)** chlorophyll content, **(D)** photosynthetic rate, **(E)** stomatal conductance, and **(F)** transpiration rate. Data are presented as mean ± SD (n = 5), and error bars represent standard deviation.

**Figure 2:**
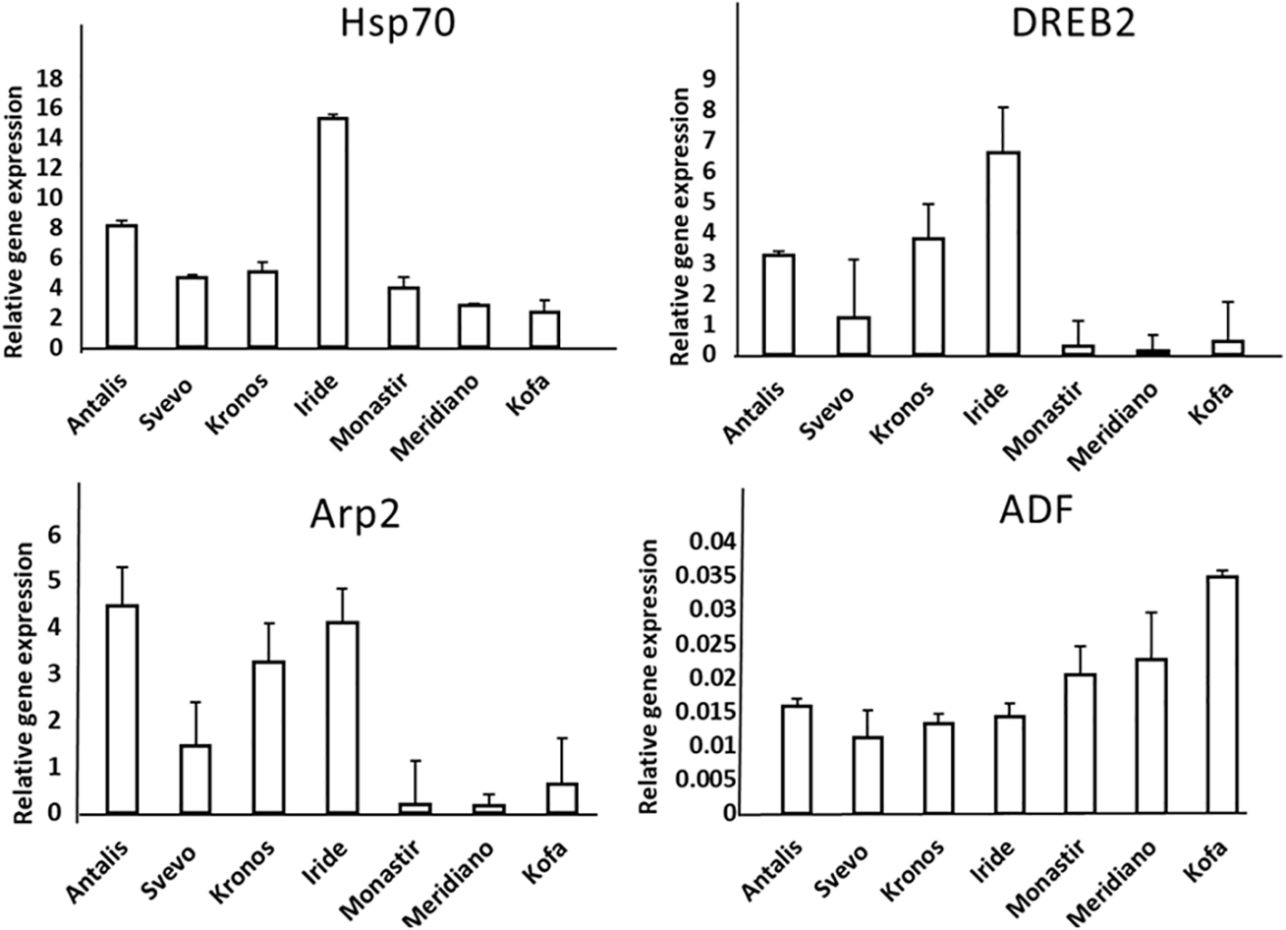
Relative expression of stress responsive genes in wild-type durum wheat varieties. Data are presented as mean ± SD (n = 5), and error bars represent standard deviation.

Seven durum wheat cultivars and four *S. bicolor* genotypes were evaluated under controlled drought stress. Significant genotype-dependent variation was observed across physiological and molecular parameters (**Figures 1**, **2**). Among durum cultivars, Kofa exhibited the highest membrane injury and the lowest relative water content (RWC) under drought stress (**Figures 1A, B**). Chlorophyll content and photosynthetic rate were also significantly reduced in Kofa compared with other cultivars (**Figures 1C, D**). In contrast, Kronos maintained the highest photosynthetic rate under drought conditions. All cultivars showed reductions in stomatal conductance and transpiration under stress (**Figures 1E, F**).

Gene expression analysis revealed strong induction of endogenous *Hsp70* in drought-tolerant cultivars, with 15-fold and 8-fold increases in Iride and Antalis, respectively (**Figure 2**). DREB2 and Arp2 expression followed similar trends, whereas Kofa exhibited comparatively weaker induction of these genes and elevated ADF expression under stress. These results indicate that Kofa displays comparatively lower drought tolerance at both physiological and molecular levels.

Among *S. bicolor* genotypes, S579 maintained superior membrane stability, RWC, and chlorophyll content under drought stress relative to other genotypes (**Supplementary Figures 1**, **3B**). Photosynthetic parameters declined in all genotypes; however, S579 consistently exhibited reduced stress-induced deterioration. Based on these observations, S579 was selected as the donor genotype for Hsp70 isolation.

### 3.2 Drought-induced formation of interlocking marginal lobes

**Figure 3:**
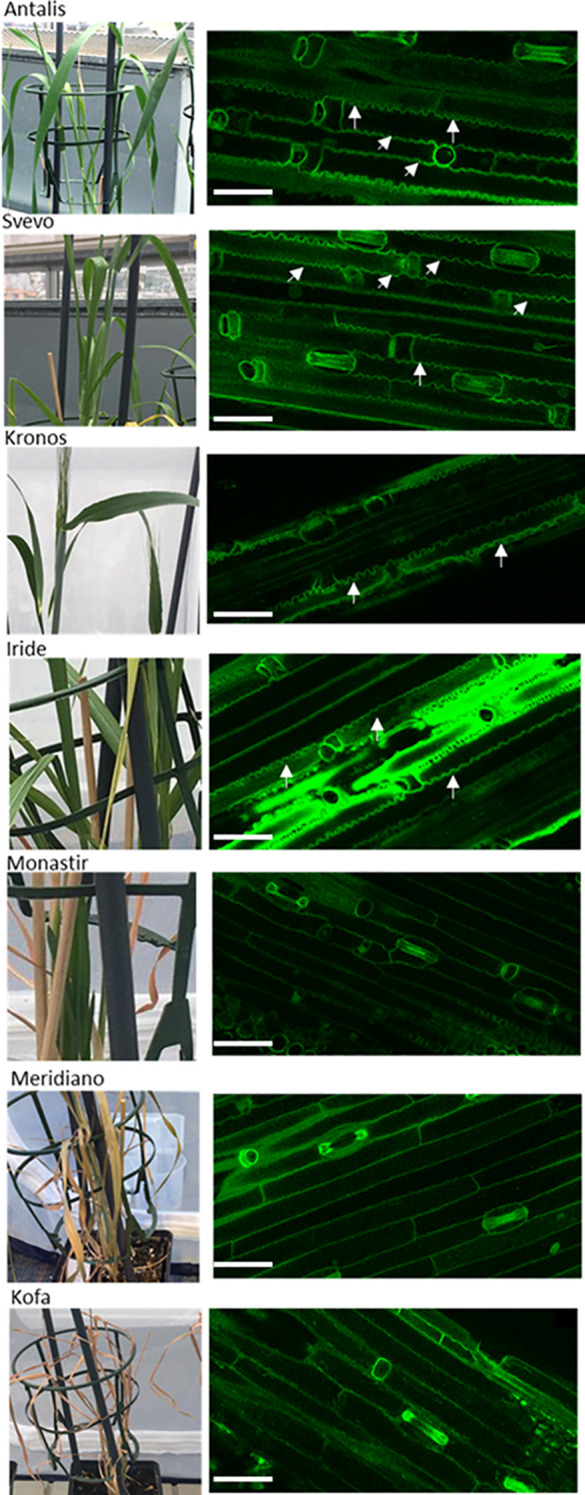
Interlocking marginal lobe (IML) formation in wild-type durum wheat varieties under drought stress. Left panels show whole-plant phenotypes of durum wheat varieties exposed to drought, and right panels show confocal images of the lower epidermal cell layers stained with Alexa Fluor 488–phalloidin to visualise IML formation. White arrows indicate the presence of interlocking marginal lobes (IMLs). Drought-tolerant varieties exhibit pronounced IML formation, whereas drought-sensitive varieties show reduced or absent IML structures. Scale bar 50µm.

Drought stress induced formation of interlocking marginal lobes (IMLs) in drought-tolerant genotypes (**Figure 3**). Cultivars exhibiting higher membrane stability displayed pronounced IML formation, whereas drought-sensitive genotypes showed limited or absent IML development. S579 exhibited early and progressive IML formation under stress (**Supplementary Figure 3**). Elevated Hsp70 expression coincided with enhanced IML formation, suggesting a relationship between stress-responsive chaperone activity and cellular structural adaptation.

### 3.3 Generation and characterization of SbHsp70 transgenic wheat

The *SbHsp70* coding sequence (2049 bp) was isolated from S579 and confirmed by sequencing (GenBank Accession No. PV482836). The sequence showed high homology (96–99.7%) with reported Hsp70 genes from related monocot species.

**Figure 4:**
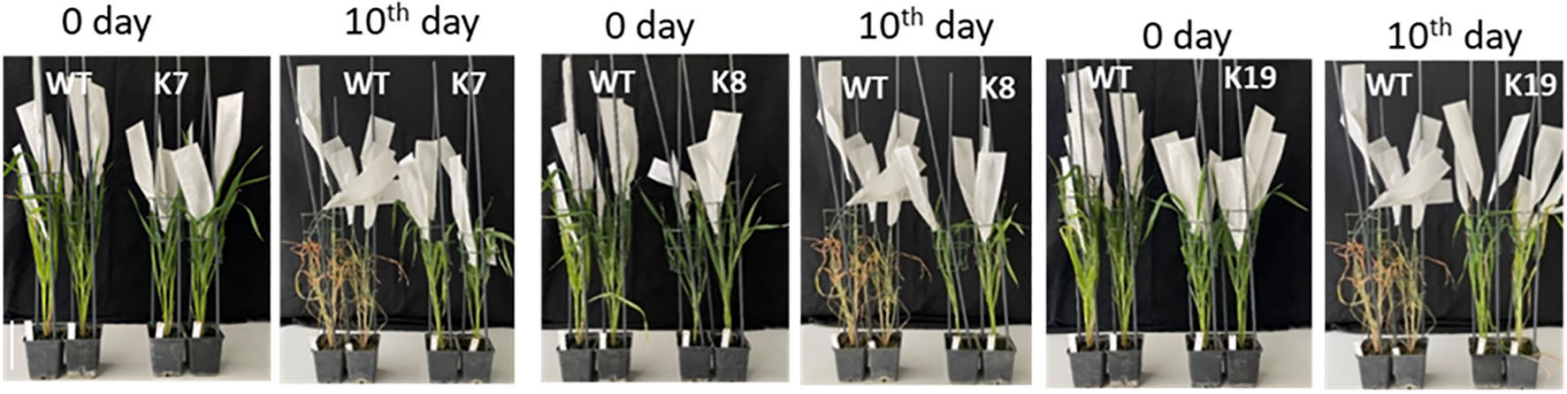
Phenotypic screening of durum wheat ‘Kofa’ plants overexpressing SbHsp70 compared with wild-type controls under drought stress. Images were taken on the 0th and 10th day of drought treatment. A vertical scale bar (10 cm) is included on the left to indicate plant height.

**Figure 5:**
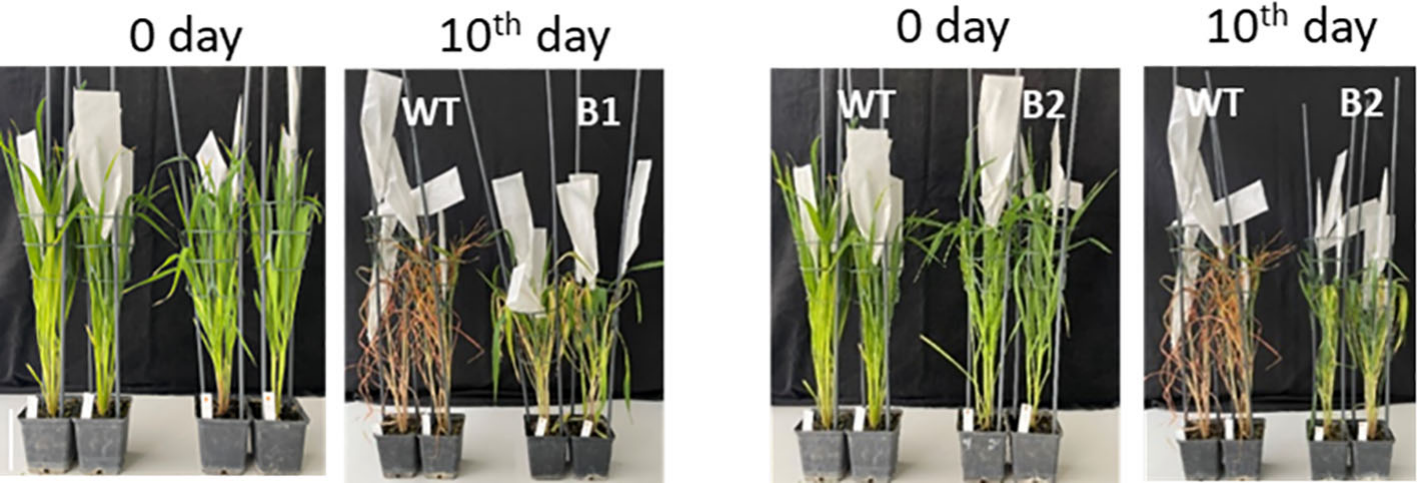
Screening of bread wheat ‘Bobwhite’ plants overexpressing SbHsp70 compared with wild-type controls under drought stress. Images were taken on the 0th and 10th day of drought treatment. A vertical scale bar (10 cm) is shown on the left to indicate plant height.

*SbHsp70* was introduced into Kofa and Bobwhite via particle bombardment. PCR screening confirmed successful integration in multiple independent events (**Supplementary Figure 5**). Under well-watered conditions, transgenic lines showed no visible morphological abnormalities, and physiological parameters were comparable to wild-type controls (**Figures 4**, **5**; **Supplementary Figure 6**), indicating absence of growth penalties.

Transgene copy number was estimated by quantitative real-time PCR (qPCR) using the endogenous reference gene *HD1*. Analysis of independent transgenic events revealed variation in transgene copy number among lines. In the tetraploid cultivar Kofa, most lines exhibited ΔCt values consistent with approximately two to three transgene copies, while a subset of lines showed estimates consistent with higher copy numbers (~4 copies).

In the hexaploid cultivar Bobwhite, the estimated copy number ranged from approximately one to three copies. Overall, most lines in both backgrounds displayed low to moderate transgene copy numbers (approximately two to three copies). Detailed estimates for all lines are provided in **Supplementary Table 5**. These values should be considered approximate, as they are based on relative qPCR quantification.

### 3.4 Morphological responses of SbHsp70-overexpressing wheat to drought stress

Under well-watered conditions, all genotypes scored 1 for wilting. After 10 days of drought stress, several transgenic Kofa (K7, K8, K19) and Bobwhite (B1, B2) events exhibited significantly reduced wilting compared with wild-type plants (**Table 1**; **Figures 4**, **5**). Wild-type plants consistently showed severe wilting (score 4).

**Table 1:** Visual scoring of *SbHsp70* overexpressed wheat varieties under drought stress.

Event number	0^th^ day	10^th^ day
K1	1	2
K2	1	2
K3	1	2
K4	1	2
K5	1	3
K6	1	2
K7	1	1
K8	1	1
K9	1	4
K10	1	4
K11	1	4
K12	1	4
K13	1	2
K14	1	3
K16	1	4
K17	1	4
K19	1	1
KWT	1	4
B1	1	1
B2	1	1
B3	1	3
B4	1	4
BWT	1	4

#### 3.4.1 Membrane stability and IML formation

**Figure 6:**
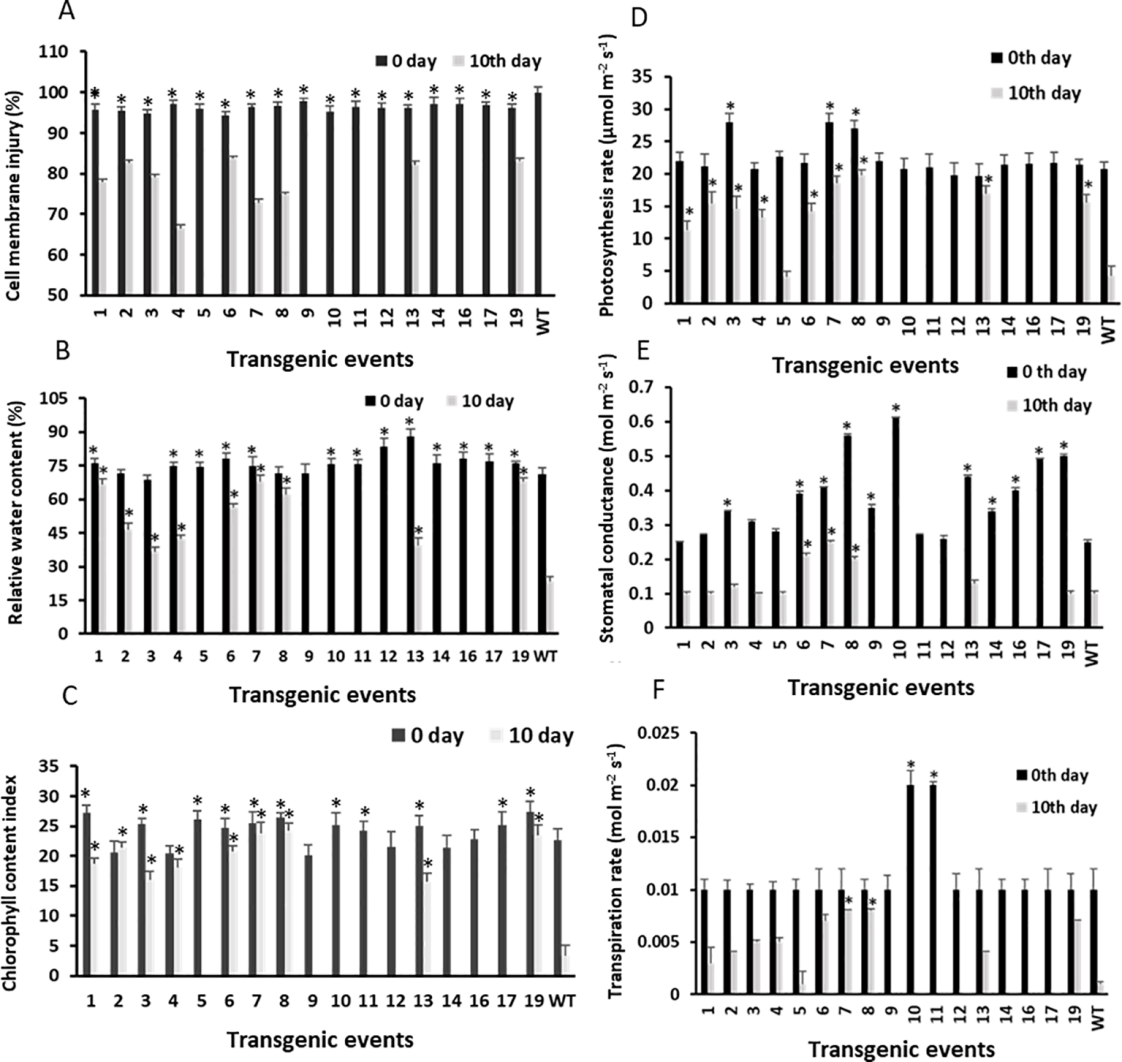
Physiological responses of durum wheat ‘Kofa’ plants overexpressing SbHsp70 compared with the wild-type control. **(A)** Cell membrane stability, **(B)** relative water content, **(C)** chlorophyll content, **(D)** photosynthetic rate, **(E)** stomatal conductance, and **(F)** transpiration rate. Significant differences between transgenic events and the untransformed control were determined using Student’s t-test (P ≤ 0.05) and are indicated by asterisks. Data are presented as mean ± SD (n = 5), with error bars representing standard deviation.

**Figure 7:**
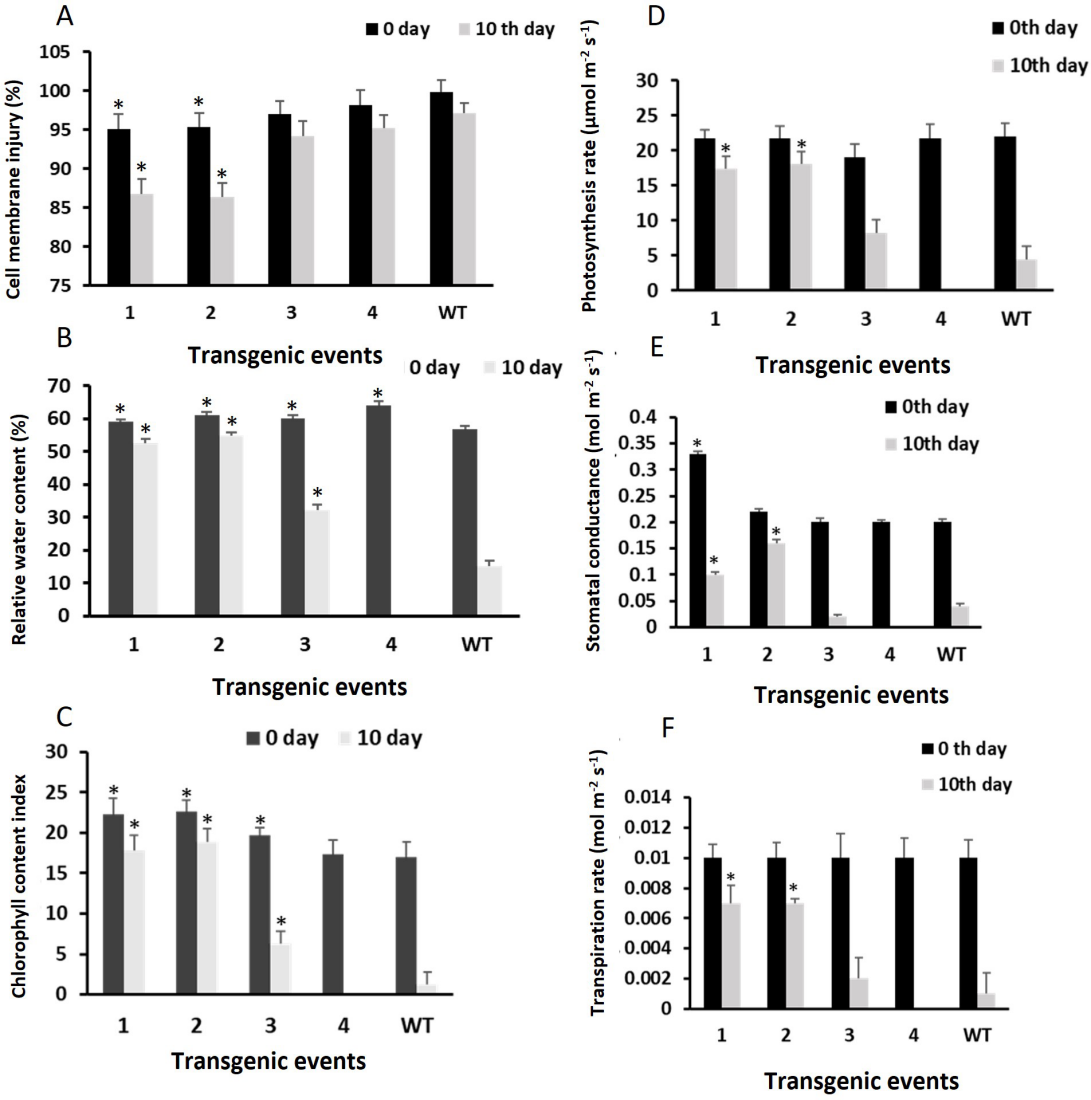
Physiological responses of bread wheat (cv. Bobwhite) plants overexpressing SbHsp70 compared with the wild-type control. **(A)** Cell membrane stability, **(B)** relative water content, **(C)** chlorophyll content, **(D)** photosynthetic rate, **(E)** stomatal conductance, and **(F)** transpiration rate. Significant differences between transgenic events and the untransformed control were determined using Student’s t-test (P ≤ 0.05) and are indicated by asterisks. Data are presented as mean ± SD (n = 5), and error bars represent standard deviation.

Cell membrane thermostability analysis demonstrated significantly reduced membrane injury in selected transgenic events under drought stress (**Figures 6A**, **7A**). K7 and K8 (Kofa) and B1 and B2 (Bobwhite) showed the highest membrane stability among evaluated lines.

**Figure 8:**
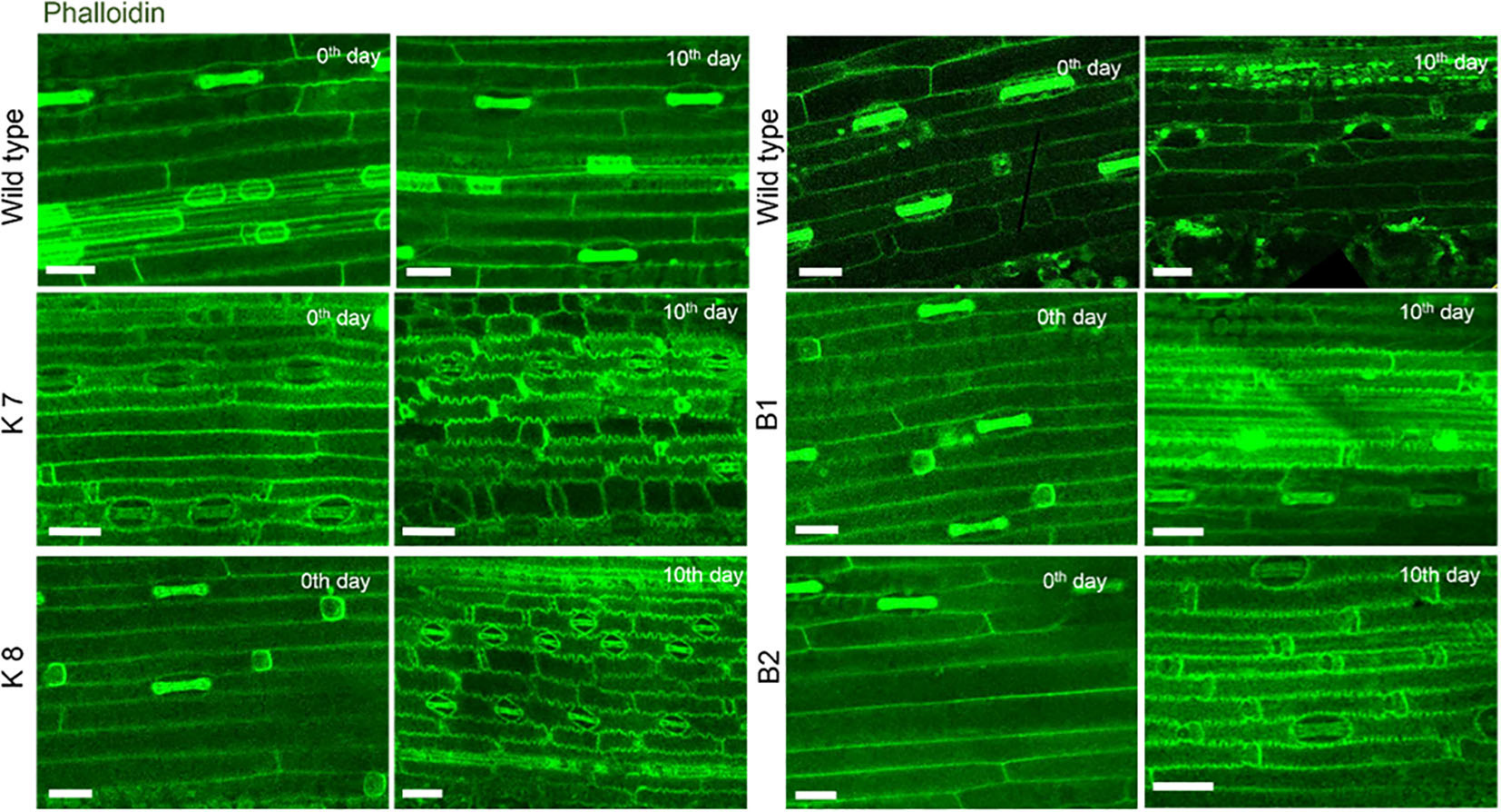
Analysis of interlocking marginal lobe (IML) formation under drought stress in Kofa and bread wheat ‘Bobwhite’ plants overexpressing SbHsp70, compared with wild-type controls. Scale bar 50µm.

Drought-induced IML formation was observed in these selected transgenic lines but was absent or less pronounced in wild-type plants (**Figure 8**), indicating enhanced cellular structural adaptation.

#### 3.4.2 Relative water content

Under drought stress, selected transgenic lines maintained significantly higher RWC compared with wild-type controls (**Figures 6B**, **7B**). K7 and K8 exhibited only minor reductions in RWC (~10%), whereas wild-type Kofa showed a 50% reduction. Similar trends were observed in Bobwhite, where B1 and B2 maintained substantially higher water status relative to wild type.

#### 3.4.3 Gas exchange and chlorophyll retention

Transgenic lines maintained significantly higher photosynthetic rate, stomatal conductance, and transpiration under drought stress compared with wild-type plants (**Figures 6D–F**, **7D–F**). Chlorophyll content declined minimally in selected transgenic events, whereas wild-type plants showed pronounced reductions (**Figures 6C**, **7C**). These results indicate improved physiological stability in SbHsp70-overexpressing lines.

### 3.5 Enhanced drought-responsive gene activation

**Figure 9:**
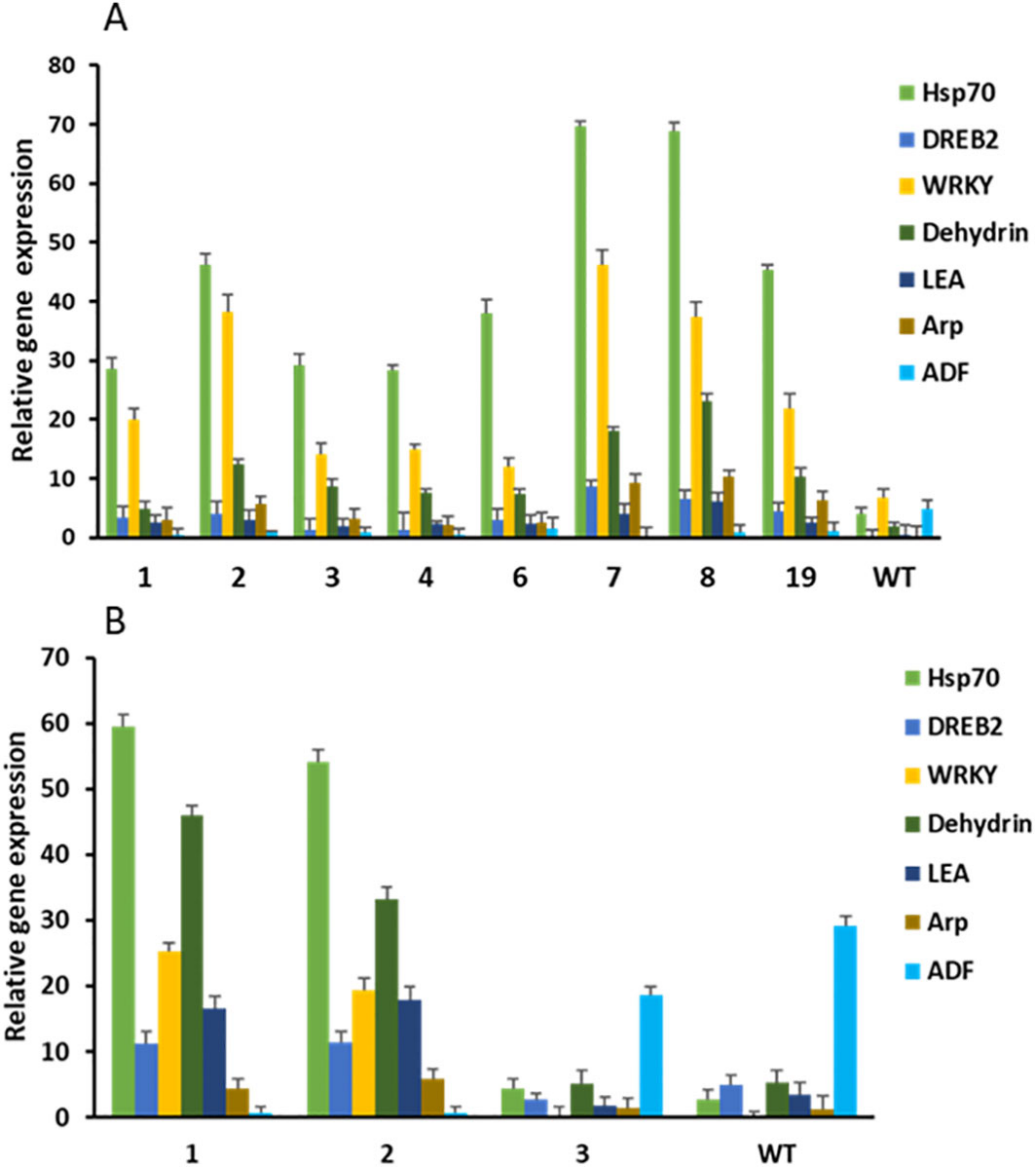
Relative expression of stress responsive genes in SbHsp70 overexpressed transgenic plants compared with wild type control plants. **(A)** Durum wheat Kofa **(B)** Bread wheat Bobwhite. Data are presented as mean ± SD (n = 5), and error bars represent standard deviation. Expression levels are shown as fold-changes relative to the respective well-watered control of each genotype (set to 1), as calculated using the 2⁻ΔΔCt method.

Under drought stress, *SbHsp70*-overexpressing lines exhibited strong upregulation of Hsp70 transcripts (up to 70-fold in K7 and K8; **Figure 9A**). In contrast, wild-type Kofa showed only a 4-fold increase.

Additionally, transgenic lines displayed enhanced expression of several drought-associated regulatory and adaptive-response genes, including DREB2, WRKY, Dehydrin, LEA, and Arp2. ADF expression was comparatively reduced in transgenic lines relative to wild type (**Figures 9A, B**). Similar expression patterns were observed in Bobwhite (**Figure 9B**).

### 3.6 Enhanced salinity tolerance during germination and early seedling development

**Figure 10:**
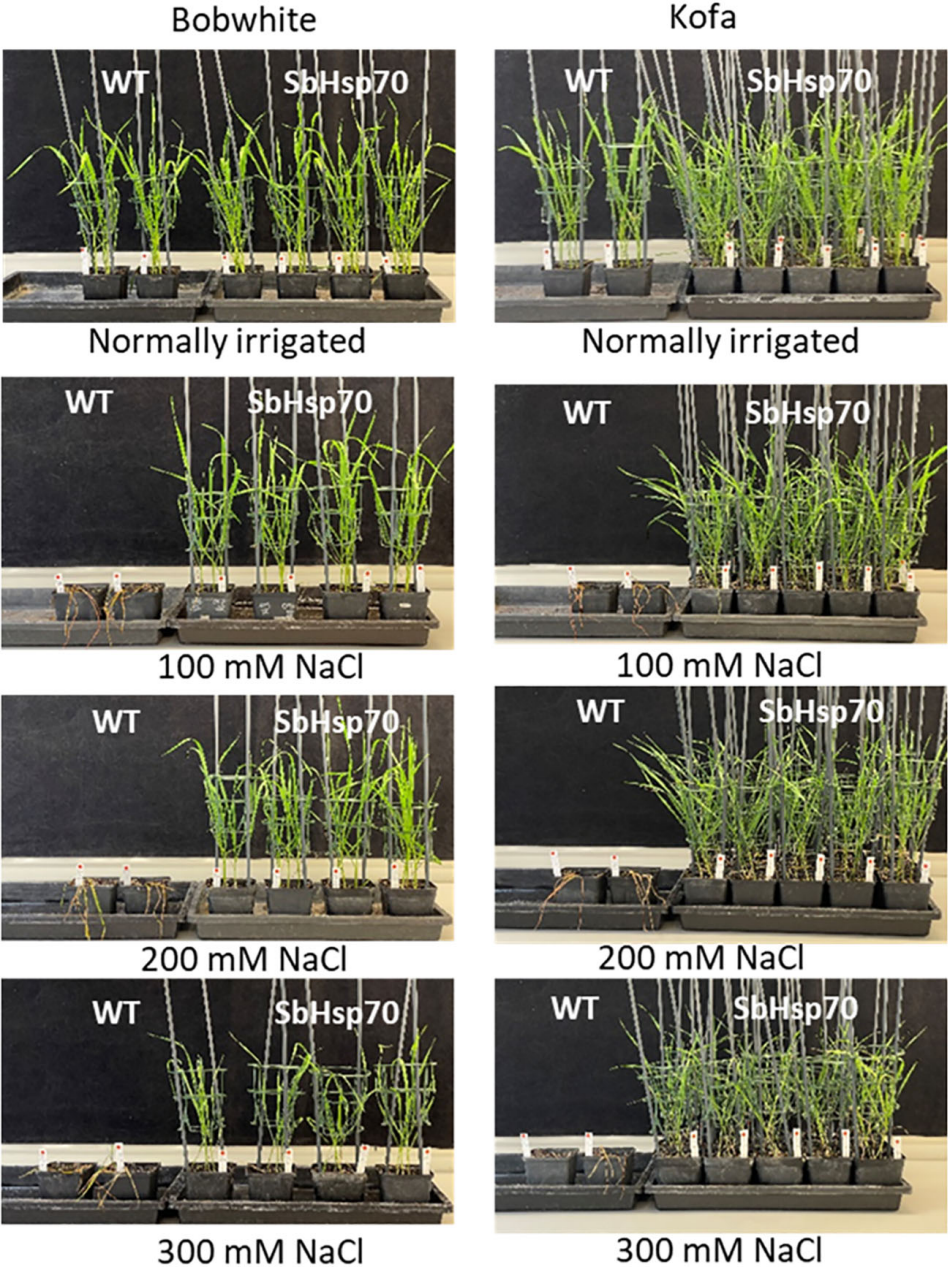
Screening for salinity tolerance using a seed germination assay in durum wheat ‘Kofa’ and bread wheat ‘Bobwhite’ plants overexpressing SbHsp70, compared with wild type controls.

Under non-saline conditions, no differences were observed between transgenic and wild-type seeds. However, increasing NaCl concentrations completely inhibited germination in wild-type controls, whereas all *SbHsp70*-overexpressing lines-maintained germination and early growth across salinity treatments (**Figure 10**), demonstrating enhanced salt tolerance.

### 3.7 Agronomic performance under precision phenotyping

DroughtSpotter analysis revealed no significant differences in yield-related traits between transgenic and wild-type plants under well-watered conditions (**Table 2**; **Supplementary Table 2**), indicating absence of yield penalty.

**Table 2:** Agronomic traits of the wild type and transgenic wheat events in the DroughtSpotter facility.

Plant lines	Water treatment	Number of spikes/containers	Number of seeds/containers	Seed weight/container (g)
K7	Well-watered	108	2982	76.98
K8	Well-watered	118	3618	74.44
KWT	Well-watered	122	2546	62.98
B1	Well-watered	136	3220	84.76
B2	Well-watered	156	2210	83.78
BWT	Well-watered	140	2274	62.66
K7	Moderate drought	73	2291	68.95
K8	Moderate drought	84	2173	48.35
KWT	Moderate drought	42	1529	42.77
B1	Moderate drought	108	2147	52.21
B2	Moderate drought	89	1333	52.34
BWT	Moderate drought	94	2287	52.00
K7	Severe drought	63	1822	53.96
K8	Severe drought	55	1719	49.29
KWT	Severe drought	72	2022	46.50
B1	Severe drought	79	1833	46.17
B2	Severe drought	66	1361	36.78
BWT	Severe drought	69	928	33.32

Under moderate and severe drought treatments, transgenic lines maintained agronomic performance comparable to wild-type controls, with no statistically significant reductions in spike number, seed number, or total seed weight. These findings confirm that SbHsp70 overexpression enhances stress resilience without compromising productivity.

## 4 Discussion

Understanding the cellular and molecular determinants of drought tolerance is critical for developing resilient wheat cultivars under accelerating climate change. In this study, we demonstrate that constitutive overexpression of *SbHsp70*, isolated from the drought-tolerant sorghum genotype S579, enhances drought and salinity tolerance in both durum wheat (Kofa) and spring wheat (Bobwhite) without compromising agronomic performance. The observed improvements in membrane stability, water status, photosynthetic performance, cytoskeletal organization, and stress-responsive gene regulation collectively provide an integrative functional framework for the role of Hsp70 in abiotic stress adaptation.

### 4.1 Membrane stability as a physiological indicator of drought tolerance

Disruption of membrane integrity is one of the earliest physiological manifestations of drought-induced cellular stress. Membrane thermostability is therefore widely used as a reliable physiological marker of stress tolerance in crops (Saneoka et al., 2004). Significant genotype-dependent variation in membrane injury has been reported in sugarcane (Venkataramana et al., 1987; Augustine et al., 2015c), maize (Coskun et al., 2011), wheat (Fokar et al., 1998), and barley (Ahmed et al., 2013), with tolerant genotypes consistently showing enhanced membrane preservation under stress. In wheat, improved membrane stability has been directly linked to superior heat tolerance and yield maintenance under elevated temperatures (Fokar et al., 1998).

In this study, *SbHsp70*-overexpressing lines consistently exhibited reduced membrane injury compared with wild-type plants, supporting a protective role of *Hsp70* under dehydration conditions. These findings are consistent with reports from transgenic potato expressing DcHsp17.7 (Ahn and Zimmerman, 2006) and sugarcane overexpressing *EaHsp70* (Augustine et al., 2015b), where enhanced membrane stability was associated with improved stress tolerance. Given the established function of *Hsp70* proteins as molecular chaperones that stabilize stress-labile proteins and membranes (Wang et al., 2004; Augustine et al., 2015b; He et al., 2018; Krausko et al., 2022), our results support a functional role for *SbHsp70* in stabilizing stress-sensitive cellular components, further studies are required to clarify the direct molecular targets involved.

Although membrane stability is widely used as a physiological marker of abiotic stress tolerance, it should not be interpreted as an isolated determinant of drought adaptation. In plants, membrane injury reflects the integrated outcome of multiple interconnected processes, including reactive oxygen species (ROS) production and scavenging, osmotic imbalance, cellular dehydration, and biomechanical constraints within tissues. Because leaves consist of multiple interacting cell types with distinct metabolic and physiological functions, stress-induced membrane damage likely represents a system-level response involving coordinated cellular and tissue-level regulation. In addition, mechanical interactions between cell wall architecture, turgor maintenance, and cytoskeletal organization may contribute to membrane-associated stress responses under dehydration conditions. Therefore, the enhanced membrane stability observed in SbHsp70-overexpressing lines should be interpreted within the broader context of integrated cellular homeostasis and whole-tissue stress adaptation rather than as a single causal determinant of drought tolerance.

### 4.2 Maintenance of water status and photosynthetic capacity

Relative water content (RWC) reflects a plant’s ability to maintain cellular hydration under water-limiting conditions and is closely linked to osmotic adjustment and cellular elasticity (Ritchie et al., 1990; Gunasekera and Berkowitz, 1992; Levinsh, 2023; Volaire et al., 2026). *SbHsp70*-overexpressing wheat lines maintained significantly higher RWC under drought stress, suggesting improved water retention capacity.

Drought-induced reductions in photosynthesis are typically driven by stomatal closure and decreased mesophyll conductance (Flexas et al., 2004; Chaves et al., 2009; Li et al., 2025). While stomatal regulation protects against excessive water loss (Chaves et al., 2003), it also restricts CO_2_ assimilation. Notably, transgenic lines maintained higher photosynthetic rates, stomatal conductance, and chlorophyll content under stress. Maintenance of chlorophyll is a key indicator of chloroplast stability and sustained photosynthetic performance (Wright et al., 1994; Nageswara Rao et al., 2001; Ahmad et al., 2021), with similar associations reported in stress-tolerant tomato lines (Camejo et al., 2005; Kavi Kishor et al., 2022). These findings indicate that SbHsp70 overexpression supports physiological resilience by sustaining carbon assimilation during water deficit.

### 4.3 Associated with cytoskeletal stabilization and interlocking marginal lobe formation

A notable observation in this study was the enhanced formation of interlocking marginal lobes (IMLs) in drought-tolerant genotypes and *SbHsp70*-overexpressing lines. These structural changes were accompanied by altered actin organization and reduced expression of actin-depolymerizing factor (ADF), suggesting a potential link between Hsp70 activity and cytoskeletal stability under stress conditions. Comparable responses have been reported in sugarcane, where Hsp70 overexpression promoted actin stabilization and improved drought tolerance (Augustine et al., 2015a).

Hsp70 proteins play central roles in proteostasis and may influence cytoskeletal organization under stress (Wang et al., 2004; Augustine et al., 2015a; He et al., 2018; Backe et al., 2025). Elevated ADF expression has previously been linked to drought sensitivity in sugarcane and tobacco (Qin et al., 2011; Augustine et al., 2015a; 2021; Tovignan et al., 2023). The observed association between *SbHsp70* overexpression, actin organization, and IML formation suggests that cytoskeletal stabilization may contribute to improved cellular resilience. However, direct mechanistic interactions between *Hsp70* and cytoskeletal components were not examined in this study and remain to be established.

### 4.4 Coordinated activation of stress-responsive pathways

Beyond structural stabilization, *SbHsp70* overexpression was associated with enhanced transcriptional activation of multiple drought-responsive genes, including DREB2, WRKY, Dehydrin, LEA, and Arp2. These genes are commonly associated with abiotic stress responses and may contribute to improved tolerance through osmotic adjustment and cellular protection.

The strong fold induction of *SbHsp70* in transgenic lines reflects its regulation by a constitutive and stress-responsive promoter (Phillip et al., 2013), while stable expression of the reference gene ensured accurate normalization. Previous studies have demonstrated that Hsp70 overexpression enhances stress signaling capacity and limits oxidative damage (Guo et al., 2007; Amudha and Balasubramani, 2011; Krishna et al., 2019; Kumar et al., 2026). The present data extend these findings by linking chaperone activity to cytoskeletal dynamics and membrane stability in wheat.

### 4.5 Proposed mechanistic model of SbHsp70-mediated stress tolerance

**Figure 11:**
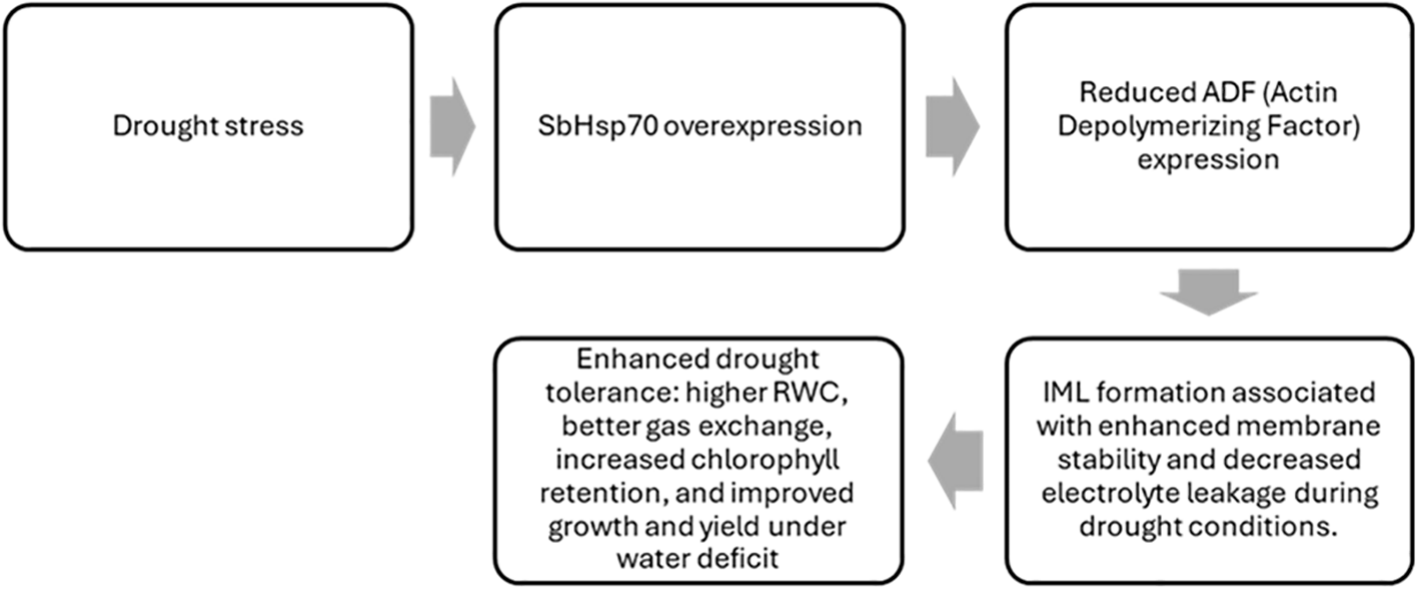
Proposed mechanistic model for SbHsp70-mediated drought tolerance in wheat.

To integrate these observations, we propose a conceptual model summarizing *SbHsp70*-mediated stress adaptation (**Figure 11**). Under drought and salinity stress, dehydration triggers protein misfolding, membrane destabilization, cytoskeletal disorganization, and oxidative imbalance. Constitutive overexpression of *SbHsp70* enhances molecular chaperone capacity, preserving proteostasis and stabilizing stress-sensitive proteins.

Concurrently, reduced ADF expression promotes F-actin stabilization and IML formation, strengthening plasma membrane–cell wall interactions and limiting ion leakage. Coordinated activation of stress-responsive genes further supports osmotic adjustment, cellular protection, and sustained photosynthetic activity. Together, these interconnected processes maintain membrane integrity, water status, and carbon assimilation under stress conditions.

### 4.6 Enhanced salinity tolerance and broad-spectrum stress resilience

In addition to drought tolerance, *SbHsp70*-overexpressing lines exhibited improved germination and survival under salinity stress. Salt stress imposes osmotic and oxidative challenges similar to drought, and *Hsp70*-mediated protection against reactive oxygen species has been documented across plant systems (Guo et al., 2007; Amudha and Balasubramani, 2011; Krishna et al., 2019; Sharma et al., 2025). The observed salt tolerance suggests that SbHsp70 acts as a central regulator of broad-spectrum abiotic stress resilience.

### 4.7 Integrative perspective and translational implications

This study advances current understanding of *Hsp70*-mediated stress adaptation by integrating physiological, structural, and transcriptional evidence into a unified mechanistic framework. While previous studies have demonstrated protective roles of Hsp70 in individual systems (Ahn and Zimmerman, 2006; Augustine et al., 2015b; Song et al., 2025), our findings extend these observations by linking *SbHsp70* overexpression to cytoskeletal reorganization, IML formation, and coordinated activation of stress-responsive networks in wheat. The association between reduced ADF expression, enhanced F-actin stability, and membrane reinforcement provides new mechanistic insight into structural adaptations underlying drought tolerance.

Importantly, the absence of yield penalties under optimal conditions addresses a major limitation often associated with constitutive stress-gene overexpression strategies. Precision phenotyping using the DroughtSpotter platform further strengthens the translational relevance of these findings by demonstrating agronomic stability under controlled drought scenarios.

Rather than defining a direct mechanistic pathway, the present findings suggest that *SbHsp70* contributes to stress adaptation through coordinated effects on cellular stability, structural responses, and stress-associated gene expression. Further studies employing targeted functional analyses will be required to elucidate the precise molecular mechanisms underlying these observations. Direct molecular interactions remain to be experimentally validated.

Although the present study demonstrates that *SbHsp70* overexpression enhances drought and salinity tolerance through improved physiological stability and stress-associated structural adaptations, abiotic stress responses in plants involve highly coordinated interactions among multiple organs, tissues, and cell types. Drought and salinity tolerance are not solely determined by bulk gene expression changes or membrane stability within leaf tissues, but rather by dynamic integration of hormonal signaling, metabolism, cellular communication, and organ-level coordination. In leaves, distinct cell populations including mesophyll, guard, vascular, and epidermal cells contribute differently to stress perception and adaptive responses. Furthermore, root-derived signals play central roles in coordinating whole-plant drought responses through hormonal and hydraulic regulation (Pasternak and Yaroshko, 2026). Since transcript analysis in this study was performed using bulk leaf tissue, the observed expression profiles represent overall tissue-level responses rather than cell-type-specific regulatory mechanisms. Therefore, the proposed role of *SbHsp70* should be interpreted within the context of integrated whole-plant stress adaptation rather than as an isolated leaf-specific response. Future studies employing cell-type-resolved transcriptomics, metabolomics, and root–shoot signaling analyses will be important for understanding the broader regulatory networks associated with *SbHsp70*-mediated stress tolerance.

The enhanced drought and salinity tolerance observed in *SbHsp70*-overexpressing wheat lines may be associated with the molecular chaperone activity of *Hsp70* proteins under abiotic stress conditions. *Hsp70* proteins are known to facilitate proper protein folding, prevent stress-induced protein aggregation, and assist in the stabilization and refolding of damaged proteins during cellular stress. Under drought and salinity stress, overexpression of *SbHsp70* may contribute to the maintenance of cellular homeostasis by protecting membrane integrity, preserving photosynthetic machinery, and supporting stress-responsive metabolic processes. The improved physiological performance and reduced cellular damage observed in the transgenic lines further suggest a possible role of *SbHsp70* in minimizing oxidative and osmotic stress-associated injury. In addition, *Hsp70*-mediated protection of cellular proteins may help sustain normal metabolic and developmental processes under stress conditions, thereby contributing to improved stress resilience without major yield penalties.

## 5 Conclusion

Collectively, this study demonstrates that constitutive overexpression of *SbHsp70* significantly enhances drought and salinity tolerance in wheat through integrated physiological, molecular, and structural mechanisms. Strengthened membrane stability, improved water retention, sustained photosynthetic capacity, coordinated stress-gene activation, and cytoskeletal reinforcement collectively contribute to enhanced stress resilience. Importantly, these benefits were achieved without yield penalties under favorable conditions. These findings position *SbHsp70* as a promising candidate for next-generation wheat improvement programs aimed at developing climate-resilient cereal crops for sustainable food production.

## Data Availability

The original contributions presented in the study are included in the article/**Supplementary Material**. Further inquiries can be directed to the corresponding author. The sequence data presented in the study are deposited in the NCBI repository, accession number PV482836.
