# Supplementary material for: SbHsp70 overexpression enhances drought and salinity tolerance in wheat through improved cellular stability and stress-associated structural adaptations
Source: Front Plant Sci. 2026 Jun 18;17:1868690. doi: 10.3389/fpls.2026.1868690 (PMC13323143; doi:10.3389/fpls.2026.1868690)
Supplement: Supplementary file 1 [file SupplementaryFile1.docx]

**Supplementary Figures**

**Figure 1**: Analysis of drought stress tolerance in different *Sorghum bicolor* genotypes. (A) Cell membrane thermostability (B) Relative water content (RWC) (C) Chlorophyll content (D) Photosynthesis rate (E) Stomatal conductance and (F) Transpiration rate. Data are presented as mean ± SD (n = 5) and error bars represent SD.


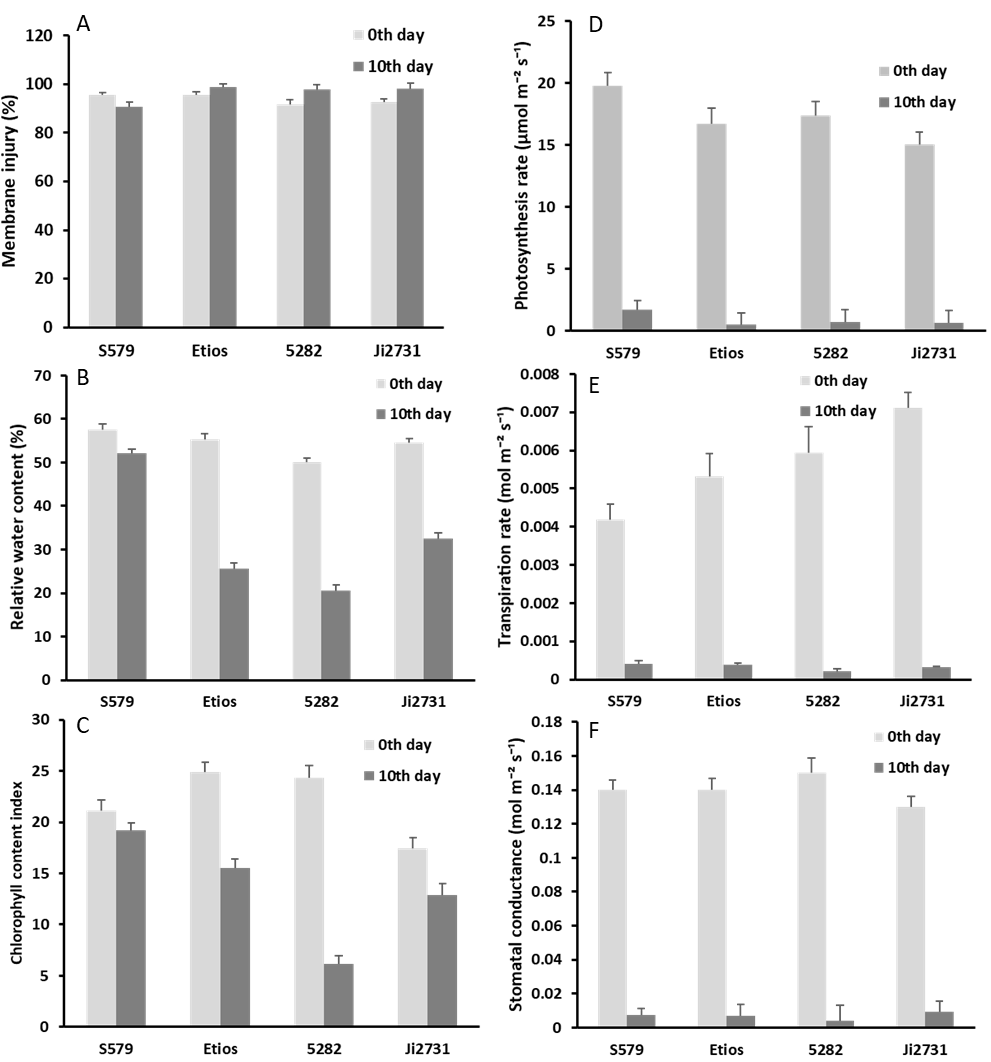


**Figure 2**: The analysis of the IML formation and membrane stability in durum wheat varieties under drought stress.


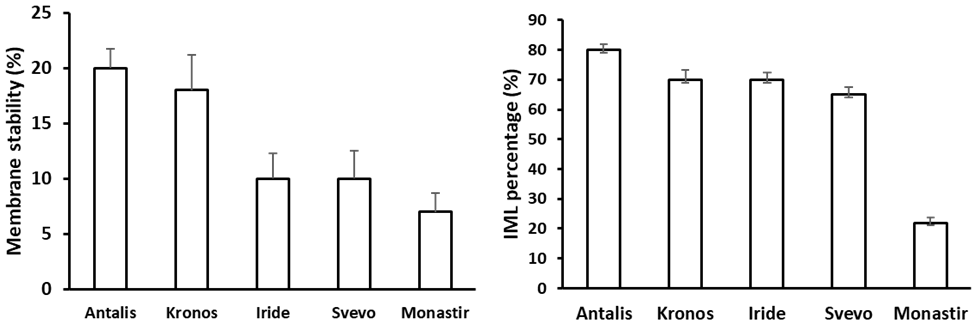


**Figure 3**: *Sorghum bicolor* genotype S579. A) Analysis of IML on the 0^th^ and 10^th^ day of stress in S579. B) Morphological screening of the S579 under drought stress.


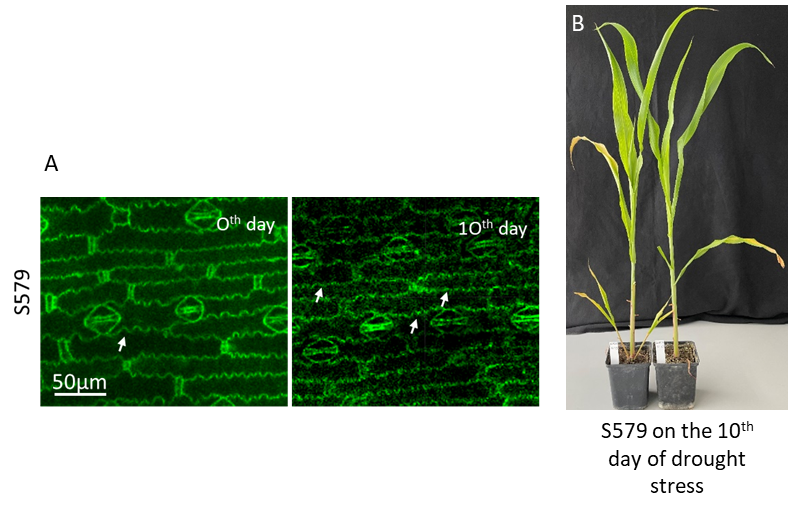


**Figure 4**: SbHsp70 gene isolated from S579


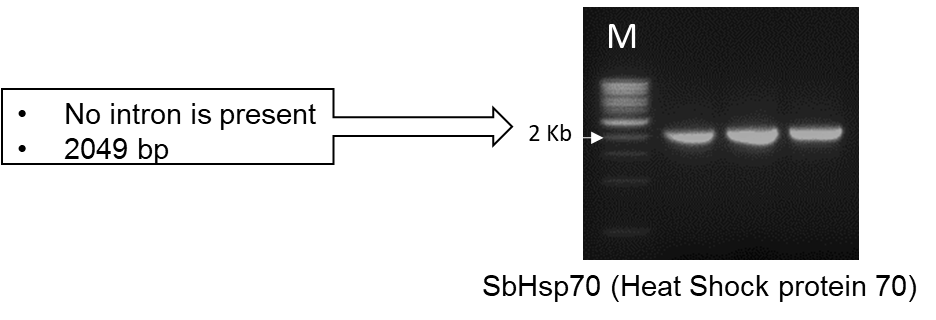


**Figure 5**: Wheat transformation. A) Construct map B) Kofa transformation C) Confirmation of the Kofa transgenic plants D) Bobwhite transformation E) Confirmation of the Bobwhite transgenic plants.


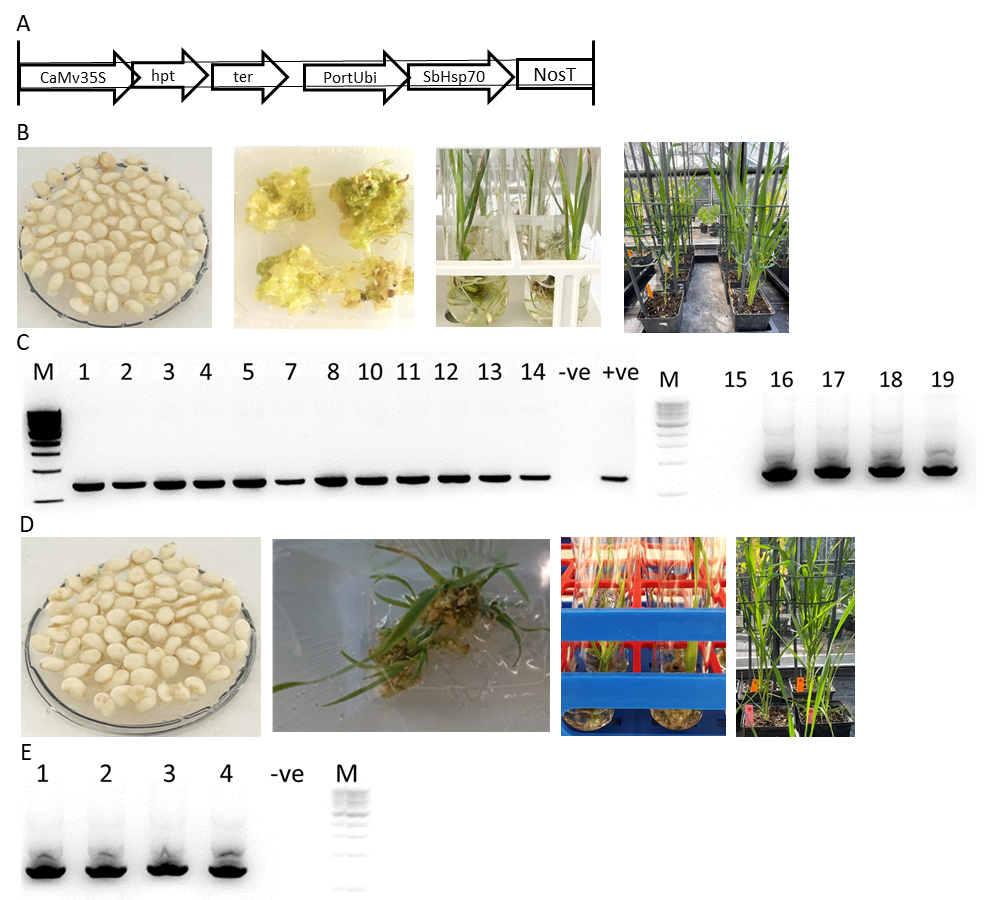


**Figure 6**: Screening of durum wheat Kofa carrying SbHsp70 overexpression compared to wild type control plants under drought stress on the 10th day of stress.

**
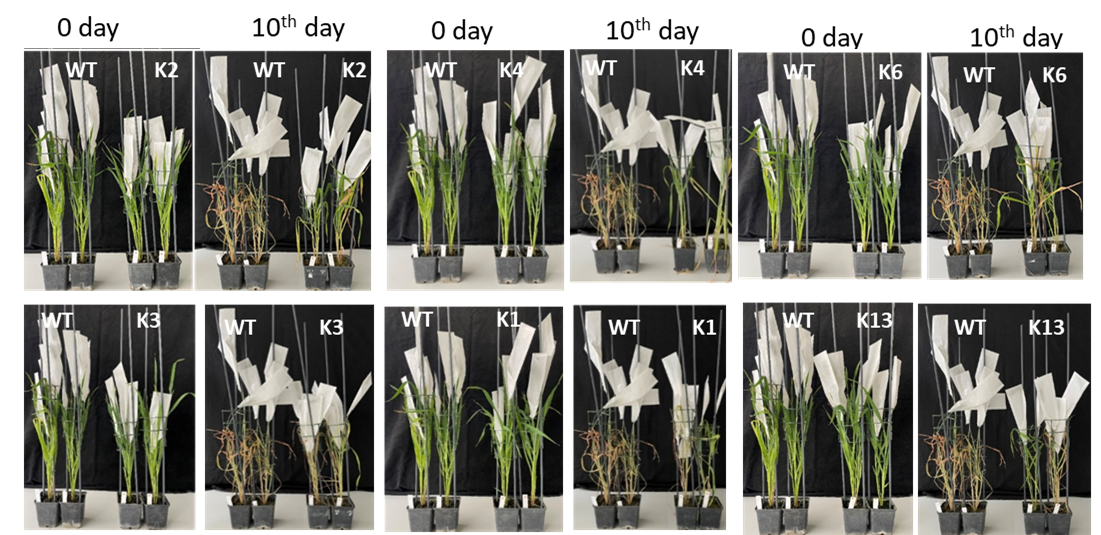
**

**Figure 7**: DroughtSpotter analysis of the SbHSP70 overexpressed transgenic and wild type plants.


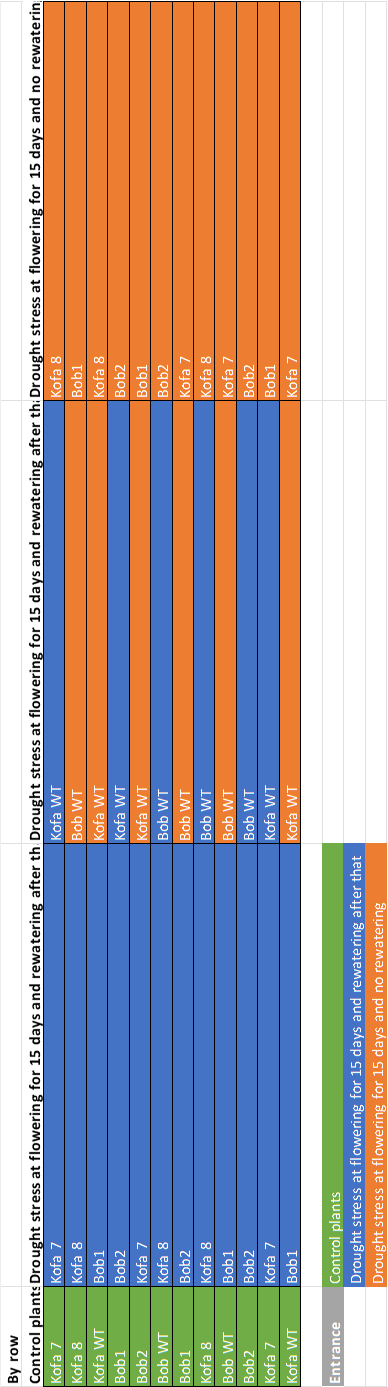


**Supplementary Table**

**Table 1:** Primer sequences

| **Name** | **Primer sequence** | **Amplicon size** |
| --- | --- | --- |
| Hsp70 F | ATGGCCGGAAAGGGAGAC | 2049 bp |
| Hsp70 R | TTAGTCGACTTCTTCGATCTT |  |
| Hpt F | GAACTCACCGCGACGTCTGTCGAG | 735 bp |
| Hpt R | GAAGTAGCGCGTCTGCTGCTCCATA |  |

**Table 2:** ANOVA summary for durum and spring wheat under three irrigation treatments

| **Species** | **Treatment** | **Trait** | **F-value** | **p-value** | **Significance** |
| --- | --- | --- | --- | --- | --- |
| Kofa | 1 | Spikes | 0.073 | 0.930 | ns |
| Kofa | 1 | Seeds | 0.571 | 0.721 | ns |
| Kofa | 1 | Seed Weight | 0.259 | 0.927 | ns |
| **Kofa** | **2** | **Spikes** | **4.008** | **0.078** | ns (trend) |
| Kofa | 2 | Seeds | 1.697 | 0.261 | ns |
| Kofa | 2 | Seed Weight | 1.862 | 0.235 | ns |
| Kofa | 3 | Spikes | 0.912 | 0.451 | ns |
| Kofa | 3 | Seeds | 0.225 | 0.805 | ns |
| Kofa | 3 | Seed Weight | 0.097 | 0.909 | ns |
| Bobwhite | 1 | Spikes | 0.083 | 0.921 | ns |
| Bobwhite | 1 | Seeds | 0.741 | 0.516 | ns |
| Bobwhite | 1 | Seed Weight | 0.405 | 0.684 | ns |
| Bobwhite | 2 | Spikes | 1.094 | 0.393 | ns |
| **Bobwhite** | **2** | **Seeds** | **3.680** | **0.091** | ns (trend) |
| Bobwhite | 2 | Seed Weight | 0.0004 | 0.9996 | ns |
| Bobwhite | 3 | Spikes | 0.543 | 0.607 | ns |
| Bobwhite | 3 | Seeds | 2.329 | 0.178 | ns |
| Bobwhite | 3 | Seed Weight | 0.629 | 0.565 | ns |

**ns = not significant (p ≥ 0.05)**

**Trend = p 0.05–0.10**

**Table 3:** qPCR Primer sequences

| **Name** | **Primer sequence** | **Amplicon size** |
| --- | --- | --- |
| Hsp70 F | TGCTGGATGTAACACCCCTT | 112 bp |
| Hsp70 R | GTGGAGAAGGTCTGGCTCTT |  |
| WRKY F | CACAAGTACGACCAGCAGTG | 145 bp |
| WRKY R | CGGTGAGGTGAGGAGCATG |  |
| ADF F | CTTCTTCATCCACTGGTCGC | 147 bp |
| ADF R | AGGATGTTGAGGGTGAGCTC |  |
| DREB2 F | TCTTTCCCGGACGTCAAGAG | 128 bp |
| DREB2 R | GGAGCCTCCGAGAAATCAAG |  |
| Arp F | CTGTGGGTGTCCTTTGCTTC | 147 bp |
| Arp R | GCTGGGCTTTTGACATGGAA |  |
| LEA F | AGTACACCAAGGAGTCCGC | 147 bp |
| LEA R | TGGTGATGGTGTTGTCCCC |  |
| ADP F | ATGCTGTGCTGCTTGTGTTT | 164 bp |
| ADP R | CCAGTCCAGGCCTTCATACA |  |
| Dehydrin F | AGTTACCGGCGAGAACATCA | 126 bp |
| Dehydrin R | GACTTCCCGTAGTTGCCATC |  |

**Table 4:** Primer sequences

| **Name** | **Primer sequence** | **Amplicon size** |
| --- | --- | --- |
| HD1F | GTGCCAAGCGTGGTGTACTG | 172 bp |
| HD1R | ACAACGCTGCAGCATCTGCAT |  |
| Promo-gene F | AATACGGAGGAGCGGTTTGA | 171 bp |
| Promo-gene R | TCGCGTGATGATGATTGTGG |  |

**Table 5:** Copy number determined using qPCR

| **Number** | **Transgenic Events** | **Copy Number** |
| --- | --- | --- |
| 1 | K1 | 2 |
| 2 | K2 | 4 |
| 3 | K3 | 3 |
| 4 | K4 | 2 |
| 5 | K5 | 1 |
| 6 | K6 | 2 |
| 7 | K7 | 4 |
| 8 | K8 | 4 |
| 9 | K9 | 1 |
| 10 | K10 | 1 |
| 11 | K11 | 4 |
| 12 | K12 | 3 |
| 13 | K13 | 2 |
| 14 | K14 | 3 |
| 15 | K16 | 2 |
| 16 | K17 | 2 |
| 17 | K19 | 4 |
| 18 | B1 | 3 |
| 19 | B2 | 3 |
| 20 | B3 | 1 |
| 21 | B4 | 1 |
